# Supplementary material for: OpenMM 7: Rapid development of high performance algorithms for molecular dynamics
Source: PLoS Comput Biol. 2017 Jul 26;13(7):e1005659. doi: 10.1371/journal.pcbi.1005659 (PMC5549999; doi:10.1371/journal.pcbi.1005659)
Supplement: S1 Source Code — (ZIP) [file pcbi.1005659.s002.zip › OpenMM-7.0.1-Source/docs/api-python/generated/simtk.openmm.openmm.Integrator.html]

Integrator — OpenMM 7.0.1.dev-5e86c4f documentation


# Integrator¶

*class* `simtk.openmm.openmm.``Integrator`(*\*args*, *\*\*kwargs*)¶
:   An Integrator defines a method for simulating a System by integrating the equations of motion. This is an abstract class. Subclasses define particular integration methods.

    Each Integrator object is bound to a particular Context which it integrates. This connection is specified by passing the Integrator as an argument to the constructor of the Context.

    `__init__`(*\*args*, *\*\*kwargs*)¶

    Methods

    |  |  |
    | --- | --- |
    | `__init__`(\*args, \*\*kwargs) |  |
    | `getConstraintTolerance`((self) -> double) | Get the distance tolerance within which constraints are maintained, as a fraction of the constrained distance. |
    | `getStepSize`((self) -> double) | Get the size of each time step, in picoseconds. |
    | `setConstraintTolerance`(self, tol) | Set the distance tolerance within which constraints are maintained, as a fraction of the constrained distance. |
    | `setStepSize`(self, size) | Set the size of each time step, in picoseconds. |
    | `step`(self, steps) | Advance a simulation through time by taking a series of time steps. |

    `getStepSize`(*self*) → double¶
    :   Get the size of each time step, in picoseconds. If this integrator uses variable time steps, the size of the most recent step is returned.

        |  |  |
        | --- | --- |
        | Returns: | the step size, measured in ps |
        | Return type: | double |

    `setStepSize`(*self*, *size*)¶
    :   Set the size of each time step, in picoseconds. If this integrator uses variable time steps, the effect of calling this method is undefined, and it may simply be ignored.

        |  |  |
        | --- | --- |
        | Parameters: | **size** (*double*) – the step size, measured in ps |

    `getConstraintTolerance`(*self*) → double¶
    :   Get the distance tolerance within which constraints are maintained, as a fraction of the constrained distance.

    `setConstraintTolerance`(*self*, *tol*)¶
    :   Set the distance tolerance within which constraints are maintained, as a fraction of the constrained distance.

    `step`(*self*, *steps*)¶
    :   Advance a simulation through time by taking a series of time steps.

        |  |  |
        | --- | --- |
        | Parameters: | **steps** (*int*) – the number of time steps to take |

    `__copy__`(*self*) → Integrator¶

    `__delattr__`¶
    :   x.\_\_delattr\_\_(‘name’) <==> del x.name

    `__format__`()¶
    :   default object formatter

    `__getattribute__`¶
    :   x.\_\_getattribute\_\_(‘name’) <==> x.name

    `__hash__`¶

    `__reduce__`()¶
    :   helper for pickle

    `__reduce_ex__`()¶
    :   helper for pickle

    `__sizeof__`() → int¶
    :   size of object in memory, in bytes

    `__str__`¶

High performance molecular simulation on GPUs


### Navigation

- Application Layer
- Library Layer
  - Core Objects
  - Forces
  - Integrators
  - Extras

©2015, Stanford University and the Authors.
|
Powered by Sphinx 1.3.1
& Alabaster 0.7.7
|
Page source
